# Clinical characteristics of eosinophilic COPD versus COPD patients with a history of asthma

**DOI:** 10.1186/s12931-017-0559-0

**Published:** 2017-04-26

**Authors:** Umme Kolsum, Arjun Ravi, Paul Hitchen, Satyanarayana Maddi, Thomas Southworth, Dave Singh

**Affiliations:** 1 Division of Infection, Immunity and Respiratory Medicine, School of Biological Sciences, Faculty of Biology, Medicine and Health, Manchester Academic Health Science Centre, The University of Manchester and University Hospital of South Manchester NHS Foundation Trust, Manchester, M23 9QZ UK; 2 The Medicines Evaluation Unit, Manchester, M23 9QZ UK

**Keywords:** Eosinophils, COPD, Asthma COPD overlap, ACOS

## Abstract

Eosinophilic COPD appears to be a distinct patient subgroup with an increased corticosteroid response. Eosinophilic COPD has been labelled as part of the asthma COPD overlap syndrome (ACOS). We compared the clinical characteristics of eosinophilic COPD patients (without any clinical history of asthma) and COPD patients with a childhood history of asthma. COPD patients with asthma were characterised by more allergies and more exacerbations, but less eosinophilic inflammation. While terms such as “ACOS” are used to “lump” patients together, we report distinct differences between eosinophilic COPD and COPD patients with asthma, and propose that these groups should be split rather than lumped.

## To the Editor:

Eosinophilic COPD appears to be a distinct patient subgroup with a corticosteroid treatment response [1, 2]. Furthermore, some, but not all, studies have shown that higher blood eosinophil counts in COPD patients predict a higher exacerbation rate in the future [3, 4]. The presence of eosinophilic inflammation in this COPD subgroup suggests that a similarity to asthma is present. Indeed, eosinophilic COPD has been labelled as part of the asthma-COPD overlap, also termed the asthma COPD overlap syndrome (ACOS) [5]. The asthma-COPD overlap consists of multiple subgroups of patients with distinct clinical and pathophysiological features, and there is a need to further define the characteristics of these subgroups [6]. We have compared the clinical characteristics of eosinophilic COPD patients without any clinical history of asthma, and COPD patients with a childhood history of asthma. The aim was to understand the similarities and differences between eosinophilic COPD and patients with a clinical diagnosis of both asthma and COPD.

Patients were recruited (Oct 2014-June 2016) from a research database of COPD patients from primary care who responded to media advertising. Two groups of patients were recruited; 67 COPD patients with no history of asthma were randomly selected from the database, and 14 COPD patients were specifically selected due to a history of childhood asthma (asthma and COPD; AC). 14 of the 67 COPD patients without asthma had a blood eosinophil count ≥300 cells/μl and were called COPD blood eosinophil^high^, while 24 patients had a sputum eosinophil count ≥3% and were called COPD sputum eosinophil^high^. All patients were aged >40 years, with post-bronchodilator forced expiratory volume in 1 second (FEV_1_)/forced vital capacity (FVC) ratio <0.7 and ≥10 pack year smoking history. COPD patients attended for a visit at stable state; defined as not experiencing an exacerbation requiring antibiotics and/or oral corticosteroids in the preceding 6 weeks. Exacerbation history in the 12 months prior to study entry was based on patient recall. All patients provided written informed consent using protocols approved by the Greater Manchester Ethics Committees (10/H/1003/108 and 05/Q1402/41). COPD patients performed the following procedures: St George’s Respiratory Questionnaire (SGRQ), COPD Assessment Test (CAT), Clinical COPD Questionnaire (CCQ), skin prick testing, fat free mass index (FFMI) assessment, FeNO_50_ measurements, spirometry, body plethysmography, transfer factor, 6 minute walk test (6MWT), and sputum induction as previously described [7]. Sputum was processed for differential cell counts and quantitative polymerase chain reaction detection of *H.influenzae*, *M.catarrhalis* and *S.pneumoniae* as previously described [8]. Blood eosinophil counts and serum immunoglobulin E (IgE) levels (upper limit of normal = 100 IU/ml) were measured. A sample size of approximately 15 patients with AC and eosinophil^high^ COPD was planned, giving 80% power to detect a difference in sputum eosinophil % of 3.8% between groups using a SD of 3.6 based on our own and published data [9]. Statistical analyses were performed using unpaired t-tests, Mann-Whitney tests or chi-square tests as appropriate using GraphPad Prism version 7.00 (San Diego, California; USA). *P* < 0.05 was considered statistically significant.

The clinical characteristics are shown in Table 1. The AC group were significantly younger (means: 57 versus 69 years; *p* < 0.0001) with an earlier age of diagnosis compared to the COPD group, although the duration of COPD after diagnosis was similar between groups. Pack year history and inhaled corticosteroid use were similar between groups. More AC patients reported chronic bronchitis compared to COPD patients (86% versus 55%, *p* = 0.04), while CAT, SGRQ and CCQ scores were similar between groups. These demographic and symptom differences between groups were also present when comparing the AC and both the blood and sputum eosinophil^high^ COPD patients, except for chronic bronchitis prevalence which was no longer different between groups. AC patients experienced more exacerbations in the previous year (median = 3) compared to the whole COPD population, or the blood and sputum eosinophil^high^ COPD subgroups (median = 1 for all groups).

**Table 1:** Differences and similarities in clinical features between the COPD population, blood eosinophil^high^ COPD subgroup and AC patients

	AC *n* = 14 Group A	COPD population *n* = 67		A vs. B *p* value	A vs. C *p* value
		COPD *n* = 67 Group B	^a^Blood Eosinophil^high^ COPD subgroup *n* = 14 Group C		
Gender (% Male)	79	67	79	0.53	>0.99
Age (years)	57.0 (6.9)	69.2 (6.7)	66.7 (8.3)	**<0.0001**	**0.002**
BMI (kg/m^2^)	29.1 (4.5)	27.0 (5.3)	27.8 (4.9)	0.18	0.47
FFMI(kg/m^2^)	16.8 (2.7)	16.8 (4.4)	17.3 (3.3)	0.98	0.69
Smoking history (% Ex)	71	78	79	0.73	>0.99
Pack years	33.7 [13.5–69.8]	44.0 [14.5–154.0]	47.1 [24.0–154.0]	0.25	0.09
Exacerbation (12mths prior)	3 [0–5]	1 [0–6]	1 [0–4]	**0.003**	**0.04**
Chronic bronchitis (%)	86	55	64	**0.04**	0.38
Any comorbidities (%)	85	84	79	>0.99	>0.99
Age COPD diagnosis	50.7 (5.9)	61.2 (7.5)	61.7 (7.5)	**<0.0001**	**0.0002**
Duration of COPD diagnosis (years)	8 [0.5–16]	8 [1–38]	7.5 [3–11]	0.52	0.94
ICS use (%)	93	78	71	0.28	0.33
ICS dose (BDP equivalent)	1550 [0–2000]	1000 [0–2000]	900 [0–2000]	0.45	0.35
SGRQ total	34.0 [15.3–78.8]	46.7 [3.2–90.5]	39.1 [3.2–90.5]	0.95	0.65
CAT	19.3 (8.8)	17.7 (8.5)	16.4 (10.1)	0.53	0.37
CCQ total	1.8 [0.6–5.1]	2.2 [0.1–4.8]	1.8 [0.1–4.1]	0.88	0.53
Skin prick positive to >1 allergen (%)	50	1.54	7.14	**<0.0001**	**0.03**
Allergic rhinitis (%)	0	4.6	7.1	>0.99	>0.99
Hayfever (%)	57.1	10.4	7.1	**0.0003**	**0.01**
Eczema (%)	35.7	11.9	21.4	0.04	0.68
Yes to do you suffer from allergies? (%)	85.7	9.0	7.1	**<0.0001**	**<0.0001**
Family history of asthma (%)	53.8	23.4	28.6	**0.04**	0.44
Total IgE (IU/ml)	156 [2–2458]	51 [2–6360]	85 [8–307]	0.17	0.43
High IgE (>100 IU/ml)	50.0	37.3	50.0	0.23	>0.99
Pre FEV_1_ (L)	1.8 (0.7)	1.3 (0.5)	1.4 (0.6)	**0.005**	0.21
Pre FEV_1_ (%)	55.3 (16.6)	49.0 (18.8)	51.8 (22.6)	0.25	0.64
Pre FEV_1_/FVC	0.5 (0.1)	0.4 (0.1)	0.4 (0.2)	**0.009**	0.10
Post FEV_1_ (L)	2.0 (0.7)	1.4 (0.6)	1.6 (0.7)	**0.003**	0.20
Post FEV_1_ (%)	61.5 (16.5)	54.4 (18.9)	57.6 (22.8)	0.19	0.60
Post FEV_1_/FVC	0.52 (0.1)	0.43 (0.1)	0.42 (0.1)	**<0.01**	**0.02**
Reversibility %	8.1 [-4.9–30.4]	9.0 [-7.6–48.2]	11.2 [-3.0–48.2]	0.96	0.67
Reversibility (ml)	175.0 [-100.0–510.0]	100 [-100–500]	130.0 [-60.0–500.0]	0.37	0.90
BDR ≥12% and 200ml (%)	28.6	22.4	35.7	0.73	>0.99
BDR ≥15% and 400ml (%)	14.3	4.5	7.14	0.20	>0.99
Raw (kPa.sec)	0.4 (0.2)	0.4 (0.2)	0.6 (0.4)	0.91	0.31
sGaw (kPa.sec)	0.4 [0.2–1.1]	0.5 [0.1–2.9]	0.4 [0.1–1.3]	0.91	0.66
VC %	89.8 [77.9–111.5]	89.2 [59.8–141.5]	91.8 [65.8–141.5]	0.88	>0.99
TLC%	106.9 (16.4)	107.8 (15.7)	110.5 (13.0)	0.85	0.59
RV %	145.3 (51.7)	141.9 (51.2)	148.0 (46.9)	0.83	0.90
IC %	82.1 (14.0)	72.8 (18.1)	68.9 (21.6)	0.09	0.10
FRC %	125.5 (26.5)	131.5 (39.8)	142.1 (30.4)	0.60	0.19
KCO %	79.2 (13.3)	76.5 (20.5)	81.9 (21.8)	0.65	0.69
6MWD(metres)	376.4 (73.8)	363.9 (104.6)	399.1 (140.8)	0.70	0.63
FeNO _50_ (ppb)	11.3 [5.2–56.0]	14.2 [3.8–42.6]	16.6 [10.2–42.6]	0.18	0.14
Blood eosinophil count (10^9^/L)	0.22 [0.10–0.53]	0.20 [0.02–1.14]	0.42 [0.31–1.14]	0.25	**0.001**
Blood eosinophil %	3.5 [0.9–7.8]	2.8 [0.3–20.7]	5.4 [3.7–20.7]	0.10	**0.002**
Blood eosinophil count ≥300 cells/μl	35.7	20.9	100	0.30	**0.0006**
Blood eosinophil >5%	14.3	13.4	64.3	>0.99	**0.02**
Sputum total cell count ×10^6^/g	3.3 [1.0–21.2]	5.9 [0.5–116.0]	6.2 [0.7–24.1]	**<0.01**	0.17
Sputum neutrophil %	68.8 [27.5–91.0]	82.8 [33.3–99.8]	74.8 [48.0–90.6]	**0.02**	0.53
Sputum macrophage %	21.2 [6.8–44.0]	11.6 [0.3–58.8]	18 [1.24–31.5]	**0.02**	0.23
Sputum eosinophil %	2.5 [0.5 -9.3]	2.6 [0–16.3]	7.0 [3.5–15.8]	0.92	**0.002**
Sputum lymphocyte %	0.3 [0.0–4.5]	0.3 [0.0–5.0]	0.1 [0.0–1.0]	0.22	0.30
Sputum epithelial cells %	2.8 [0.3–27.5]	0.9 [0.0–13.0]	2.1 [0.0–13.0]	**0.02**	0.30
Sputum neutrophil cell count ×10^6^/g	2.5 [0.3–17.5]	4.7 [0.3–112.5]	4.6 [0.4–21.8]	**0.01**	0.15
Sputum macrophage cell count ×10^6^/g	0.6 [0.2–2.1]	0.8 [0.0–5.3]	0.58 [0.23–1.74]	0.76	0.78
Sputum eosinophil cell count ×10^6^/g	0.1 [0.01–1.3]	0.1 [0.0–1.3]	0.4 [0.1–1.3]	0.30	**<0.01**
Sputum lymphocyte cell count ×10^6^/g	0.01 [0.0–0.06]	0.0 [0.0–0.48]	0.0 [0.0–0.08]	0.98	0.82
Sputum epithelial cell count ×10^6^/g	0.15 [0.01–0.92]	0.05 [0.0–1.35]	0.07 [0.0–0.89]	**0.03**	0.34
Sputum eosinophil ≥3%^b^	46.2	36	100	>0.99	**0.008**
Eosinophilic/Mixed/Neutrophilic/Paucigranulocytic (%)	8/33/ 31/23	8/40/ 46/6	30/70/ 0/0	**<0.0001**	**<0.0001**
Bacterial load (genome copies/ml)	4.97×10^4^ [0.00–2.06×10^6^]	7.55×10^4^ [0.00–4.01×10^7^]	5.52×10^4^ [0.00–9.33×10^6^]	0.41	0.77
Colonised (sum bacterial load ≥1×10^4^) (%)	83	65	64	0.31	0.37

Data is presented as mean (SD), medians [range] or % as appropriate; spirometry values were related to the reference values of the European Community for Coal and Steel (ECCS)

The bold *p* values represent significant *p* values

^a^14 out of the 67 COPD patients (Group B) with no history of asthma had blood eosinophil count ≥300 cells/μl (Eosinophil^high^, Group C)

^b^50 patients from the COPD population produced a sputum sample

*Definitions of abbreviations: AC* COPD with childhood asthma diagnosis, *ICS* Inhaled corticosteroids, *BDP* Beclometasone disproportionate, *BMI* Body Mass Index, *FFMI* Fat Free Mass Index, *BDR* Bronchodilator response, *Raw* Airway resistance, *sGAW* specific conductance, *SGRQ* St George’s Respiratory Questionnaire, *CCQ* Clinical COPD Questionnaire, *CAT* COPD Assessment Test, *Pre* Pre bronchodilator, *Post* Post bronchodilator, *FEV*_*1*_ Forced Expired Volume in first second, *FVC* Forced vital capacity, *VC* Vital Capacity, *TLC* Total Lung Capacity, *RV* Residual Volume, *IC* Inspiratory Capacity, *FRC* Functional Residual Capacity, *KCO* Carbon monoxide transfer coefficient, *6MWD* 6 minute walk distance, *FeNO*_*50*_ Fractional exhaled nitric oxide at 50ml/sec flow rate

Focusing on the comparison of AC and blood eosinophil^high^COPD patients, the self-reported prevalence of allergies, including hayfever, and the presence of positive skin prick tests were higher in the AC group, although the total IgE count was similar between groups. The post-bronchodilator FEV_1_ % predicted was not different between groups, but the FEV_1_/FVC ratio was higher in the AC group (mean: 0.52 vs. 0.42, *p* = 0.02). There were no differences between groups for other pulmonary function tests including lung volumes and reversibility, 6MWD or FeNO_50_.

Blood eosinophil counts were higher in the blood eosinophil^high^ COPD patients; only 35.7% of AC patients had blood eosinophil counts ≥300 cells/μl (*p* = 0.0006), and median eosinophil counts were lower in AC patients (medians: 0.42×10^9^/L vs. 0.22×10^9^/L, *p* = 0.001). Sputum eosinophil counts, both as a percentage (median: 7.0 vs. 2.5, *p* = 0.002) and absolute count (median: 0.4 vs. 0.1, *p* < 0.01), were higher in blood eosinophil^high^ COPD compared to the AC patients respectively. Additionally, all patients in the blood eosinophil^high^ group had sputum eosinophil counts ≥3% (100%) compared to the AC group (46.2%), *p* = 0.008. There were no other differences between groups for sputum cell counts. Patients from each group were classified according to sputum cell counts into neutrophilic, eosinophilic, mixed granulocytic and paucigranulocytic using neutrophil and eosinophil thresholds of 61% and 3% respectively as previously described [10]. The COPD blood eosinophil^high^ patients were classified as either eosinophilic (30%) or mixed (70%), AC patients had a more even spread between the four possible groups (Table 1). Total airway bacterial load was similar between the groups.

Comparison of sputum eosinophil^high^ COPD and AC patients (Table 2) showed similar findings to those observed between blood eosinophil^high^ COPD and AC patients; there was a higher prevalence of allergy in AC patients. In addition, AC patients had a significantly higher pre- and post-bronchodilator FEV_1_ L, FEV_1_ % predicted and FEV_1_/FVC ratios compared to sputum eosinophil^high^ COPD patients. Sputum eosinophil % (medians: 6.0 versus 2.5, *p* = 0.0004) and absolute counts (medians: 0.1×10^6^/g versus 0.04 ×10^6^/g) were higher in sputum eosinophil^high^ COPD patients compared to AC patients respectively, with only 46.2% of AC patients having a sputum eosinophil count ≥3% (*p* = 0.0002). There were no differences in blood eosinophil counts between the two groups.

**Table 2:** Differences and similarities in clinical features between sputum eosinophil^high^ and AC patients

	AC *n* = 14	Sputum Eosinophil^high^ subgroup *n* = 24	*p* value
Gender (% Male)	79	75	>0.99
Age (years)	57.0 (6.9)	68.3 (7.9)	**<0.0001**
BMI (kg/m^2^)	29.1 (4.5)	27.1 (4.5)	0.19
FFMI(kg/m^2^)	16.8 (2.7)	17.2 (3.7)	0.70
Smoking history (% Ex)	71	58	0.51
Pack years	33.7 [13.5–69.8]	46.3 [16.8–154.0]	0.06
Exacerbation (12mths prior)	3 [0–5]	1 [0–5]	**0.02**
Chronic bronchitis (%)	86	71	0.44
Any comorbidities (%)	85	79	>0.99
Age COPD diagnosis	50.7 (5.9)	62.7 (7.7)	**<0.0001**
Duration of COPD diagnosis (years)	8 [0.5–16.0]	5.5 [2.0–17.0]	0.71
ICS use (%)	93	79	0.38
ICS dose (BDP equivalent)	1550 [0–2000]	1000 [0–2000]	0.60
SGRQ total	34.0 [15.3–78.8]	51.5 [6.0–90.5]	0.34
CAT	19.3 (8.8)	18.9 (9.9)	0.90
CCQ total	1.8 [0.6–5.1]	2.5 [0.5–4.7]	0.74
Skin prick positive to >1 allergen (%)	50	0	**0.0003**
Allergic rhinitis (%)	0	8.7	0.52
Hayfever (%)	57.1	12.5	**0.008**
Eczema (%)	35.7	25.0	0.71
Yes to do you suffer from allergies? (%)	85.7	8.3	**<0.0001**
Family history of asthma (%)	53.8	36.4	0.48
Total IgE (IU/ml)	156 [2–2458]	69.5 [2–1868]	0.30
High IgE (>100 IU/ml)	50.0	71.4	0.74
Pre FEV_1_ (L)	1.8 (0.7)	1.2 (0.4)	**0.003**
Pre FEV_1_ (%)	55.3 (16.6)	44.8 (14.4)	**0.048**
Pre FEV_1_/FVC	0.5 (0.1)	0.3 (0.1)	**<0.001**
Post FEV_1_ (L)	2.0 (0.7)	1.3 (0.4)	**0.003**
Post FEV_1_ (%)	61.5 (16.5)	51.0 (14.1)	**0.04**
Post FEV_1_/FVC	0.5 (0.1)	0.4 (0.1)	**<0.001**
Reversibility %	8.1 [-4.9–30.4]	15.7 [0–48.2]	0.15
Reversibility (ml)	175.0 [-100.0–510.0]	125.0 [-60.0–400.0]	0.87
BDR ≥12% and 200ml (%)	28.6	37.5	0.73
BDR ≥15% and 400ml (%)	14.3	4.2	0.54
Raw (kPa.sec)	0.4 (0.2)	0.6 (0.2)	0.11
sGaw (kPa.sec)	0.4 [0.2–1.1]	0.4 [0.1–1.2]	0.30
VC %	89.8 [77.9–111.5]	88.9 [65.8–129.5]	0.60
TLC%	106.9 (16.4)	112.3 (17.0)	0.38
RV %	145.3 (51.7)	151.1 (64.5)	0.79
IC %	82.1 (14.0)	67.4 (12.3)	**0.004**
FRC %	125.5 (26.5)	138.9 (48.5)	0.37
KCO %	79.2 (13.3)	75.6 (19.8)	0.56
6MWD (metres)	376.4 (73.8)	330.4 (95.1)	0.16
FeNO _50_ (ppb)	11.3 [5.2–56.0]	14.3 [7.0–40.3]	0.25
Blood eosinophil count (10^9^/L)	0.22 [0.10–0.53]	0.25 [0.06–1.14]	0.37
Blood eosinophil %	3.5 [0.9–7.8]	3.4 [0.9–20.7]	0.97
Blood eosinophil >5%	14.3	25.0	0.68
Blood eosinophil count ≥300 cells/μl	35.7	41.7	>0.99
Sputum total cell count ×10^6^/g	3.3 [1.0–21.2]	5.9 [0.5–33.0]	**0.04**
Sputum neutrophil %	68.8 [27.5–91]	77.8 [48.0–92.0]	0.14
Sputum macrophage %	21.2[6.8–44.0]	14.8 [1.2–32.3]	**0.03**
Sputum eosinophil %	2.5 [0.5–9.3]	6.0 [3.2–16.3]	**0.0004**
Sputum lymphocyte %	0.3 [0.0–4.5]	0.0 [0.0–1.0]	0.06
Sputum epithelial cells %	2.8 [0.3–27.5]	1.0 [0.0–13.0]	0.08
Sputum neutrophil cell count ×10^6^/g	2.5 [0.3–17.5]	4.7 [0.3–29.4]	**0.04**
Sputum macrophage cell count ×10^6^/g	0.6 [0.2–2.1]	0.6 [0.1–3.9]	0.67
Sputum eosinophil cell count ×10^6^/g	0.1 [0.01–1.3]	0.04 [0.02–1.3]	**0.001**
Sputum lymphocyte cell count ×10^6^/g	0.01 [0.0–0.06]	0.0 [0.0–0.17]	0.29
Sputum epithelial cell count ×10^6^/g	0.2 [0.0–0.9]	0.07 [0.0–0.89]	0.08
Sputum eosinophil ≥3%	46.2	100	**0.0002**
Bacterial load (genome copies/ml)	4.97×10^4^ [0.00–2.06×10^6^]	5.52×10^4^ [0.00–1.24×10^7^]	0.66
Colonised (sum bacterial load ≥1×10^4^) (%)	83	67	0.44

Data is presented as mean (SD), medians [range] or % as appropriate; spirometry values were related to the reference values of the European Community for Coal and Steel (ECCS)

*Definitions of abbreviations: AC* COPD with childhood asthma diagnosis, *ICS* Inhaled corticosteroids, *BDP* Beclometasone disproportionate, *BMI* Body Mass Index, *FFMI* Fat Free Mass Index, *BDR* Bronchodilator response, *Raw* Airway resistance, *sGAW* specific conductance, *SGRQ* St George’s Respiratory Questionnaire, *CCQ* Clinical COPD Questionnaire, *CAT* COPD Assessment Test, *Pre* Pre bronchodilator, *Post* Post bronchodilator, *FEV*_*1*_ Forced Expired Volume in first second, *FVC* Forced vital capacity, *VC* Vital Capacity, *TLC* Total Lung Capacity, *RV* Residual Volume, *IC* Inspiratory Capacity, *FRC* Functional Residual Capacity, *KCO* Carbon monoxide transfer coefficient, *6MWD* 6 minute walk distance, *FeNO*_*50*_ Fractional exhaled nitric oxide at 50ml/sec flow rate

The bold *p* values represent significant *p* values

We observed that AC patients are younger with more allergic symptoms, but less evidence of eosinophilic inflammation compared to both blood and sputum eosinophil^high^ COPD patients. These differences were associated with a higher rate of exacerbations in AC patients. While terms such as “ACOS” may be used to “lump” patients together, we report distinct differences between eosinophilic COPD and AC patients, and propose that these groups should be split rather than lumped.

The most notable clinical difference between AC patients and both the blood and sputum eosinophil^high^ COPD patients was the exacerbation rate. Similar findings have previously been reported comparing ACOS and COPD patients [11, 12]. The higher exacerbation rate may be linked to allergic mechanisms in AC patients that are not present in eosinophil^high^ COPD patients. The degree of eosinophilic airway inflammation was lower in AC patients compared to both the blood and sputum eosinophil^high^ COPD patients. The different nature of airway inflammation in these two groups further underscores that these are distinct subgroups that should not be lumped.

Interestingly, AC patients had less severe airflow obstruction measured by FEV_1_/FVC ratio; this is difficult to explain given the similar smoking histories of the patients. A contributing factor may be the age difference between the groups, as the FEV_1_/FVC ratio is known to decrease with age [13]. The younger age of the AC patients has been noted in other studies of the asthma COPD overlap [12, 14], and may represent a tendency to diagnose COPD earlier in patients with previous asthma.

Bronchodilator reversibility was similar between the AC and both the blood and sputum eosinophil^high^ COPD groups. There was also no difference between AC and the whole COPD population; these findings agree with previous studies comparing bronchodilator reversibility in ACOS and COPD patients [15, 16]. Bronchodilator reversibility has been proposed as a distinguishing ACOS feature, but it is well known that COPD patients can display marked bronchodilator reversibility [17].

The sample size of this study was relatively limited for the AC and eosinophil^high^ COPD groups, and therefore we may have missed some important differences between groups due to insufficient statistical power. However, by studying subgroups of COPD, we have reduced inter-patient variability for some endpoints, such as eosinophil counts, for which we were able to demonstrate large differences between groups. The small sample size prevented us from performing more complex statistical analysis, but nevertheless provides an initial insight into the differences between these patient groups.

The prevalence of eosinophil^high^ COPD using a blood eosinophil count ≥300 cells/μl was 21%, while using a sputum eosinophil threshold of 3% gave a prevalence of 36%. Similarly, Watz et al [18], in a post hoc analysis of 2420 COPD patients reported a prevalence of 20% when using a blood eosinophil count threshold ≥300 cells/μl while studies have reported a prevalence of up to 40% when using a sputum eosinophil count threshold of ≥3% [9, 19]. The prevalence of eosinophilic COPD clearly varies with the threshold used, but these commonly used thresholds suggest that approximately 20–40% of the COPD population could be classified as eosinophilic. Our primary aim was to compare eosinophil^high^ COPD with AC, and we have shown that only a proportion of AC patients (46%) have eosinophilic airway inflammation, in contrast to blood or sputum eosinophil^high^ COPD patients who all have eosinophilic airway inflammation.

We differ from other ACOS studies by including only patients with a childhood history of asthma. The diagnosis of asthma may also be given to COPD patients in adult life; this is often an incorrect diagnosis given to individuals on the basis of a younger age at the time of COPD diagnosis. We excluded these patients with co-diagnosis given in adult life, in order to be certain of a correct previous asthma diagnosis in the AC group.

In conclusion, our findings demonstrate that eosinophilic COPD patients have distinct characteristics compared to COPD patients with a history of asthma. AC patients are characterised by the presence of allergies and more exacerbations, but less evidence of eosinophilic inflammation. These data support the concept that different subgroups exist within the asthma COPD overlap, and should be carefully characterised [6].

## Abbreviations

6MWT: 6 minute walk test
ACOS: Asthma COPD overlap syndrome
CAT: COPD assessment test
CCQ: Clinical COPD Questionnaire
COPD: Chronic obstructive pulmonary disease
FeNO_50_: Fractional exhaled nitric oxide at 50ml/sec flow rate
FEV_1_: Forced expiratory volume in 1 second
FFMI: Fat free mass index
FVC: Forced vital capacity
IgE: Immunoglobulin E
SGRQ-C: St George’s respiratory questionnaire
